# A descriptive analysis of the indications for caesarean section in mainland China

**DOI:** 10.1186/s12884-014-0410-2

**Published:** 2014-12-12

**Authors:** Yajun Liu, Guanghui Li, Yi Chen, Xin Wang, Yan Ruan, Liying Zou, Weiyuan Zhang

**Affiliations:** Beijing Obstetrics and Gynecology Hospital, Capital Medical University, Beijing, China

**Keywords:** Caesarean section rate, Caesarean section on maternal request, Indications for caesarean section, Mainland Chinese population

## Abstract

**Background:**

In recent decades we have observed a remarkable increase in the rate of caesarean section (CS) in both developed and developing countries, especially in China. However, the real reasons for this phenomenon are uncertain. Notably, the number of women requesting elective CS without accepted valid medical indication has also increased, generating a nationwide debate because several studies have shown that this may be the underlying cause of the increase in CS rates observed recently. Therefore, we carried out a multicentre, large-sample, cross-sectional study to describe the CS rate and indications for CS in mainland China during 2011.

**Methods:**

This was a multicentre, large-sample, cross-sectional study of women who delivered infants in 39 hospitals in 14 provinces in China during 2011. We selected 111, 315 deliveries that occurred during 2011, excluding miscarriages or termination of pregnancy before 28 gestational weeks.

**Results:**

The overall rate of CS in mainland China was 54.90%. The most common indication for CS was caesarean delivery on maternal request (CDMR; 28.43%), followed by cephalo-pelvic disproportion (14.08%), fetal distress (12.46%), previous CS (10.25%), malpresentation and breech presentation (6.56%), macrosomia (6.10%) and other indications (22.12%). CDMR accounted for 15.53% of all the deliveries and 28.43% of all CS deliveries in mainland China.

**Conclusions:**

CDMR appears to be a considerable driver behind the increasing CS rate in mainland China. The relaxation of China’s “one-child policy” may translate into a greater number of CS because of previous CS delivery. To decrease the CS rate, we should first decrease the rate of CS on maternal request. Appropriate policies and guidelines should be considered to accomplish the goal.

**Electronic supplementary material:**

The online version of this article (doi:10.1186/s12884-014-0410-2) contains supplementary material, which is available to authorized users.

## Background

There has been a remarkable increase in the rate of caesarean section (CS) in both developed and developing countries in the past decades, increasing from about 5% in developed countries in the early 1970s to more than 50% in some regions of the world in the late 1990s [1-4]. Based on a survey by the World Health Organization (WHO) on methods of delivery during the period 2007–8, the rates of CS in China and other Asian countries were 46% and 27%, respectively [5], despite the fact that in 1985, WHO recommended that no region should have a CS rate over 10–15% [6,7]. This situation exists not only in China and Asia, but also in many other countries in Latin America and the Caribbean [8]. The real reason for this remarkable increase is unknown. Therefore, a widespread debate on the reasons for the progressively increasing rate of CS is taking place in both the medical and lay press. In recent years, an increasing number of women requested delivery by elective CS without a valid “medical indication” was observed because of the fear of episiotomies, long and painful labor, pelvic floor trauma and subsequent incontinence associated with vaginal birth [9]. This has been termed “caesarean delivery on maternal request (CDMR)”, and has generated worldwide debate because several studies have shown that this phenomenon may be one of the drivers of the rising CS rate [9-12]. The situation seems to be particularly striking in China, where the rate of CS can be as high as 46%, even up to 80% in some hospitals [5,13]. The WHO study showed that China had the highest CS rate (46%) as well as the highest rate of CS without medical indication [5].

The indications for CS also vary by regions and patient ethnicity [14]. To date, the real reason for the increased CS rate remains unclear, and only a few studies have reported the actual medical indications accounting for the large rise in the CS rate, especially in China [14]. Furthermore, the studies only presented a small number of deliveries from one hospital or from a local area. With the relaxation of China’s “one-child policy”, an increasing number of women can have more than one child. However, because of the remarkable rate of CS, it is probable that many women will require CS because of a previous CS. The aim of our study was to estimate the overall CS rate in mainland China, and to describe the factors associated with the increased CS rate in mainland China.

## Methods

### Ethics approval

This study was approved by the human ethics committees of the Beijing Obstetrics and Gynecology Hospital and the Capital Medical University. The names of the institutional review boards that approved the study in the other 38 hospitals were listed in the additional file 1.

### Study design

**Figure 1:**
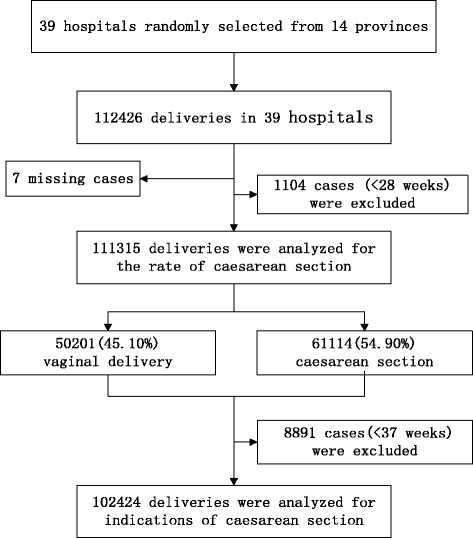
**The step-by-step description of the analysis of data.**

This was a multicentre, large-sample, cross-sectional study. To reflect the population of China, we chose hospitals in seven territories of mainland China (except Hong Kong and Macau), based on stratified random sampling: north, south, east, west, northeast, northwest, and central China. The study population comprised women who gave birth in 39 hospitals in 14 provinces in China, from 1 January 2011 to 31 December 2011. The data were obtained from 14 provinces, municipalities, and autonomous regions within China (Beijing, Shanghai, Jilin, Liaoning, Jiangsu, Sichuan, Shanxi, Hubei, Guangdong, Hebei, Inner Mongolia, Shandong, Shanxi, and Xinjiang), covering 39 hospitals of different levels. The hospitals comprised 8 secondary care and 12 tertiary care general hospitals and 12 secondary care and 7 tertiary care specialty hospitals (in the Chinese hospital classification system, tertiary care is the most specialized and primary care is the least specialized). The sample size was calculated using the following formula: N = deff u^2^ *P*(1-P) /d^2^, where deff is the design effect; N, the sample capacity; u, 1.96 when confidence coefficient is 95%; P, the probability value. According to the formula, the sample size in each layer should be 2,400. The study randomly selected 39 different hospitals of different levels of care in 14 provinces and 7 territories in mainland China. For our analysis, we selected 111,315 deliveries that occurred during the year 2011, excluding incomplete data and miscarriages or termination of pregnancy before 28 weeks of gestation because of fatal malformations, intrauterine death, or other reasons. Figure 1 shows the step-by-step description of the data collection. Gestational age was determined by the mother’s last menstrual period, and it was confirmed by an ultrasound examination within 20 weeks of gestation or by the first trimester ultrasound measurement of the crown-rump length of the fetus.

### Data collection

Questionnaire: The questionnaire included maternal characteristics; gestational, intrapartum, delivery, postpartum and neonatal care, and laboratory tests. Abstracted data included demographic data; gravidity; parity; maternal medical history; specific information on maternal or fetal pregnancy-related complications; gestational age at delivery; method of delivery; all primary indications for CS; the newborn’s sex, birth weight, birth length and Apgar score; and the maternal and perinatal outcomes. The primary indications for CS were divided into three categories: maternal indications, fetal indications, maternal request with no obstetric reasons. Maternal indications include previous caesarean delivery, elderly primigravida, cephalo-pelvic disproportion, prolonged labor (dystocia), maternal infection, complications of pregnancy such as preeclampsia, oligohydramnios, placenta praevia, placental abruption, presence of cardiac disease, or other maternal pathologies. Fetal indications included precious infant, malpresentation, fetal distress, macrosomia and multiple fetuses. The questionnaire was designed by obstetric and statistical experts and finalized after many discussions regarding its feasibility.

Training of investigators: The head of each sub-center in each province, municipality, or autonomous region accompanied 2–3 investigators while attending face-to-face training on the questionnaire entry and completion. Instructions for completing the questionnaire were also sent out to the investigators.

Data entry: Investigators from each province, municipality, and autonomous region were responsible for training their personnel for data entry. Data were collected and recorded by specially trained medical staff (obstetrics and gynecology specialists and students). Data were first entered in a hardcopy format and then entered into computer network databases.

Data collection: Data were collected and entered into a computer network database. Case collection and hardcopy data entry were carried out from January to April 2012. Then, data were entered into network database from May to June 2012, and data quality control was carried out during the same period. Each participating hospital was responsible for its own case collection and data entry, and all personnel that participated in data entry received training beforehand. Data included birth outcomes of each hospital throughout 2011.

Quality control: In each sub-center one or two specialized personnel were trained in data quality control, and they were responsible for their entire region. After the data were sent to the survey headquarters, specialized personnel at the headquarters were responsible for the second round of quality control assessment.

### Statistical analyses

All data were input into SPSS software (v.19.0; SPSS Inc., Chicago, IL, USA) for statistical analysis. Continuous variables were expressed as the mean ± SD or the median (and interquartile range), as appropriate. Differences in the baseline characteristics between two groups were tested using the Student’s t-tests for variables with normal distribution and the Mann–Whitney U tests for variables with skewed distributions. Categorical data were expressed as frequency (percentage) and the differences in frequency between the two groups were examined using the chi-squared test. A two-tailed *P*-value of <0.05 was considered significant.

## Results

In total, 111,317 deliveries at ≥28 weeks of gestation were included in the present study. Based on this sample, the overall rate of CS in mainland China was 54.90%.

**Figure 2:**
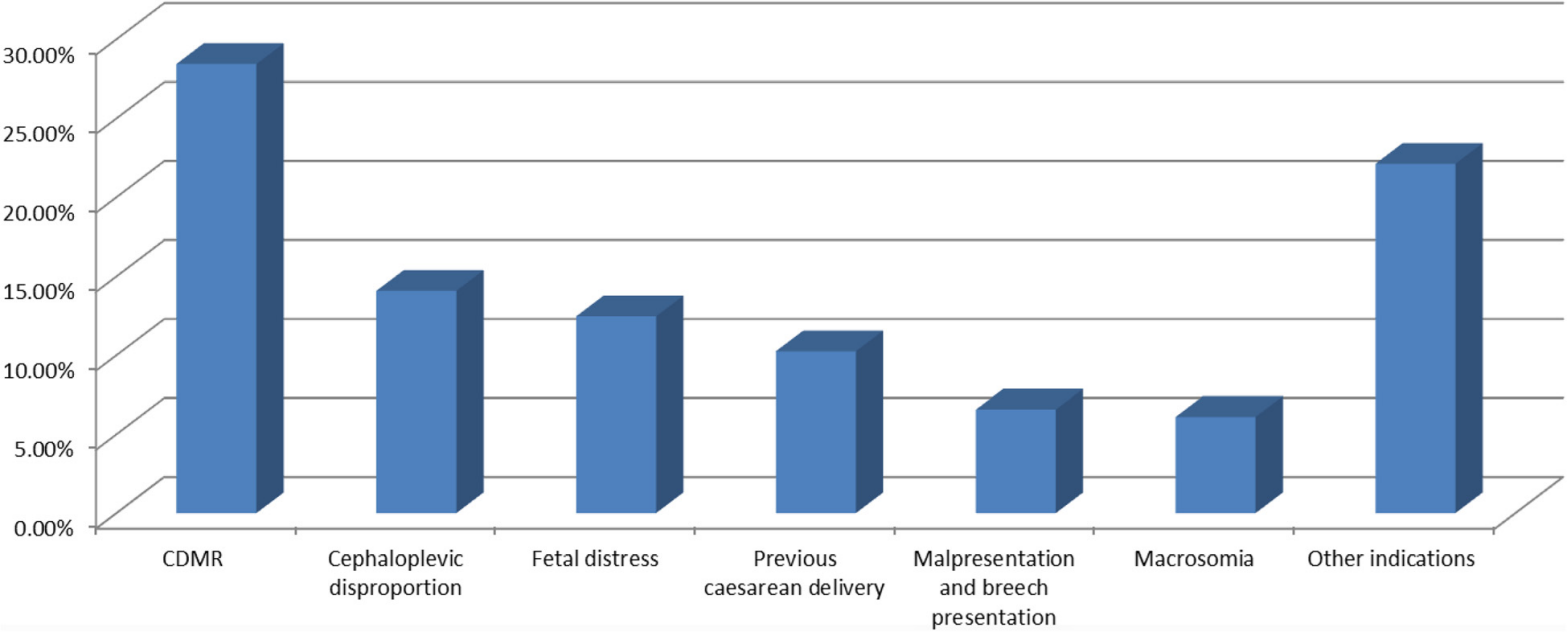
**The five main indications for caesarean section in mainland China.**

We selected only the deliveries occurring at ≥37 weeks of gestation (n = 102,424) for the analysis of the indications for CS. As shown in Figure 2, the most common indication for CS was maternal request (28.43%), followed by cephalo-pelvic disproportion (14.08%), fetal distress (12.46%), previous CS delivery (10.25%), malpresentation and breech presentation (6.56%), macrosomia (6.10%) and “other indications” (22.12%). The other indications included prolonged labor (dystocia) (3.6%), oligohydramnios (3.2%), elderly primigravida (2.4%), preeclampsia (2.1%), precious infant (1.8%) and others. In our study we identified additional indications for CS, such as multiple fetuses (0.95%), umbilical cord being wrapped around the neck (0.69%), premature rupture of membrane (0.53%), high myopia (0.47%), combined myoma of uterus and ovarian tumors (0.36%) and gestational diabetes mellitus (0.34%). CDMR accounted for 15.53% of all deliveries and 28.43% of the CS deliveries in mainland China.

Table 1 shows that the overall rate of CS in tertiary care hospitals was slightly higher than that in secondary care hospitals (56.04% vs 51.69%, *χ*^2^ = 166.59, *P* < 0.001). To some extent the distribution of the indications for CS was different between the two types of hospitals (Table 1). Although the most common indication for CS was maternal request in both secondary and tertiary care hospitals, the rate of CDMR in secondary care hospitals was higher than in tertiary care hospitals (40.15% vs 23.59%, *χ*^2^ = 1,554.71, *P* < 0.001). The rate of CS for high-risk pregnancy was higher in the tertiary care hospitals than that in the secondary care hospitals, and the indications included previous CS delivery (10.71% vs 8.70%, *P* < 0.001), malpresentation and breech presentation (7.33% vs 5.91%, *P* < 0.001), oligohydramnios (3.47% vs 2.44%, *P* < 0.001) and preeclampsia (2.52% vs 1.11%, *P* < 0.001). In contrast, the rate of CS as a result of cephalo-pelvic disproportion was higher in secondary care than that in tertiary care hospitals (16.64% vs 13.01%, *P* < 0.001), which might have been due to a lower standard of medical technology in the secondary care setting compared with the tertiary care setting. Therefore, many cephalo-pelvic disproportion cases cannot be actively managed through the stages of labor in secondary care hospitals. When we compared the rate of CS for high-risk pregnancy between the two types of hospitals, we found that the rate of high-risk pregnancies between the two types of hospitals was different. As shown in Table 2, the rate of high-risk pregnancy was higher in the tertiary care hospitals than that in secondary care hospitals, as illustrated by the frequency of the following conditions: premature rupture of membrane (17.9% vs 9.43%, P < 0.001), premature delivery (9.19% vs 2.20%, P < 0.001), gestational diabetes mellitus (6.30% vs 0.91%, P < 0.001), pregnancy-induced hypertension (6.52% vs 2.58%, P < 0.001), fetal distress (9.17% vs 6.07%, P < 0.001), intrahepatic cholestasis of pregnancy (1.51% vs 0.50%, P < 0.001), heart disease during pregnancy (0.49% vs 0.08%, P < 0.001), placenta previa (1.57% vs 0.41%, P < 0.001), placental abruption (0.69% vs 0.19%, P < 0.001).

**Table 1:** **Indications for caesarean section between different levels of hospitals in mainland China**

	**Type of hospital care**		***p***
	**Secondary**	**Tertiary**	
All deliveries (n = 102424)	31433	70991	–
CS deliveries n (%)	16248 (51.69)	39786 (56.04)	<0.001
CDMR n (%)	6523 (40.15)	9387 (23.59)	<0.001
Cephalopelvic disproportion n (%)	2703 (16.64)	5178 (13.01)	<0.001
Fetal distress n (%)	1993 (12.27)	4981 (12.52)	–
Previous CS n (%)	1413 (8.70)	4263 (10.71)	<0.001
Malpresentation and breech presentation n (%)	961 (5.91)	2918 (7.33)	<0.001
Macrosomia n (%)	752 (4.63)	2452 (6.16)	<0.001
Other indications n (%)	1903 (11.71)	10607 (26.66)	<0.001

CDMR, caesarean delivery on maternal request; CS, caesarean section.

**Table 2:** **Complications of pregnancy between different levels of hospitals in mainland China**

	**Type of hospital care**		***p***
	**Secondary**	**Tertiary**	
Premature rupture of membrane n (%)	3243 (9.43)	13771 (17.90)	<0.001
Premature delivery n (%)	757 (2.20)	7072 (9.19)	<0.001
GDM n (%)	314 (0.91)	4844 (6.30)	<0.001
PIH n (%)	887 (2.58)	5081 (6.52)	<0.001
Fetal distress n (%)	2089 (6.07)	7057 (9.17)	<0.001
ICP n (%)	173 (0.50)	1160 (1.51)	<0.001
Heart disease during pregnancy n (%)	26 (0.08)	375 (0.49)	<0.001
Placenta previa n (%)	140 (0.41)	1205 (1.57)	<0.001
Placental abruption n(%)	66 (0.19)	530 (0.69)	<0.001

GDM, Gestational Diabetes Mellitus; PIH, Pregnancy-induced hypertension; ICP, Intrahepatic Cholestasis of Pregnancy.

**Figure 3:**
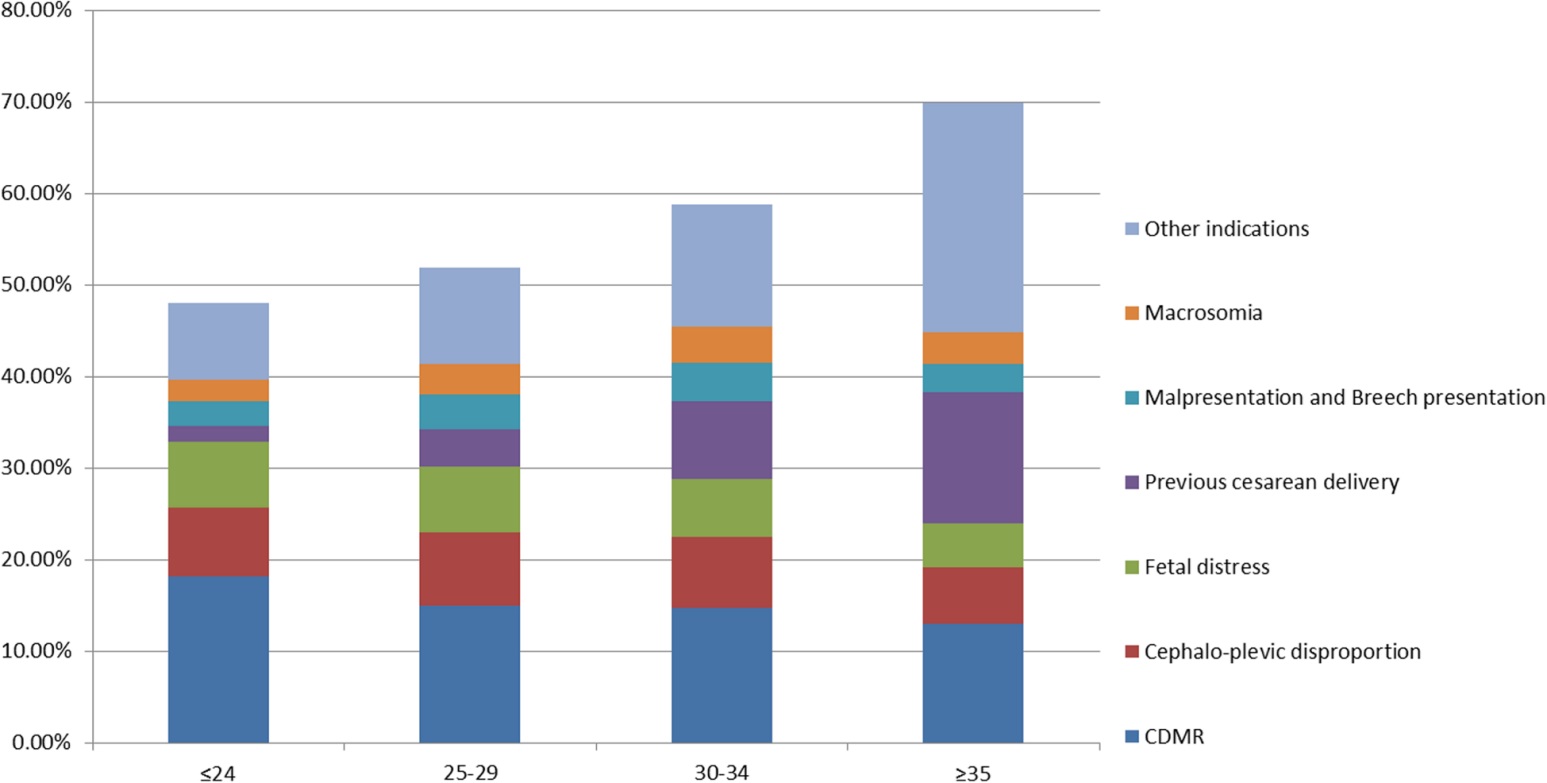
**Indications for caesarean section in different age groups.**

As shown in Table 3, the rate of CS also increased with increasing maternal age, while the rate of vaginal delivery decreased (*P* < 0.001). However, the rate of CDMR decreased with increasing maternal age. This suggests that as maternal age increased, the medical indications for CS also increased, and thus the rate of CDMR was lower (*P* < 0.001). It should be noted that there was also a key difference in the rate of previous CS delivery in different age groups (Figure 3); the rate of CS due to previous CS increased with maternal age.

**Table 3:** **Characteristics of the study populations**

		**All**	**Vaginal delivery**		**Overall rate of CS,** ***n*** **(%)**	**Caesarean section**		***P***
			**Spontaneous,** ***n *** **(%)**	**Operative,** ***n*** **(%)**		**With medical indication,** ***n*** **(%)**	**Without medical indication n (%)**	
Maternal age, years	≤24	23403	12005 (51.30)	142 (0.61)	11256 (48.10)	7011 (29.96)	4245 (18.14)	<0.001
	25–29	43661	20118 (46.08)	580 (1.33)	22663 (51.91)	16135 (36.96)	6528 (14.95)	
	30–34	25155	9992 (39.72)	387 (1.54)	14776 (58.74)	11072 (44.02)	3704 (14.72)	
	≥35	9729	2856 (29.36)	7 (0.76)	6799 (69.88)	5541 (56.95)	1258 (12.93)	
Pre-labour BMI	<18.5	107	62 (57.94)	4 (3.74)	41 (38.32)	12 (11.21)	29 (27.10)	<0.001
	18.5–24.9	25059	13507 (53.9)	319 (1.27)	11233 (44.83)	3178 (12.68)	8055 (32.14)	
	25–29.9	50802	22775 (44.83)	676 (1.33)	27347 (53.83)	7026 (13.83)	20321 (40)	
	≥30	15991	5045 (31.55)	146 (0.91)	10799 (67.53)	2174 (13.60)	8625 (53.94)	
Previous births, *n*	0	91208	39677 (43.50)	1242 (1.36)	50282 (55.13)	35306 (38.71)	14976 (16.42)	<0.001
	1	19077	9038 (47.38)	89 (0.47)	9950 (52.16)	8418 (44.13)	1532 (8.03)	
	≥2	2140	1025 (47.90)	10 (0.47)	1105 (51.64)	957 (44.72)	148 (6.92)	
Sex of child	Male	55620	23830 (42.84)	706 (1.27)	31084 (55.89)	22349 (40.18)	8735 (15.70)	<0.001
	Female	46804	21441 (45.81)	482 (1.03)	24881 (53.16)	17706 (37.83)	7175 (15.33)	

BMI, body mass index (mother’s).

Based on the pre-labor BMI of gestating women, we found that obese women had a higher rate of CS and CDMR. The rate of spontaneous vaginal delivery decreased with increasing pre-labor BMI. Additionally, nulliparous women had higher rates of CS and CDMR than parous women (*P* < 0.001) (Table 3). Male infants were more likely to be delivered by CS than female infants (55.89% vs 53.16%, *P* < 0.001). Male infants were also associated with a higher rate of operative intervention in vaginal deliveries than female infants (e.g., vacuum and forceps) (2.88% vs 2.25%; *P* < 0.001). There was no correlation between the sex of infant and the rate of CDMR, but male infants had a higher rate of delivery by CS with indications compared with female infants (40.18% vs 37.83%; *P* < 0.001).

## Discussion

Our study showed that the overall rate of CS in mainland China was 54.90% and the most common indication for CS was maternal request (28.43%). CDMR accounted for 15.53% of all deliveries and 28.43% of the CS deliveries in mainland China in our study.

The overall rate of CS in tertiary care hospitals was slightly higher than that in secondary care hospitals, probably because women with high-risk pregnancies were more likely to be admitted to tertiary care than to secondary care hospitals. This presumption is supported by the higher rate of high-risk pregnancy in the tertiary care hospitals compared with the secondary hospitals, as shown in Table 2. The distribution of the indications for CS was also somewhat different between the two types of hospitals.

Our study results also indicated that male infants were more likely than female infants to be delivered by CS. Male infants also had a higher rate of operative intervention in vaginal deliveries (e.g., vacuum and forceps) than female infants. Although there was no correlation between the sex of infants and rate of CDMR, male infants had a higher rate of delivery by CS with indications compared with female infants. This might be because male infants have higher risks of adverse perinatal complications, such as gestational diabetes mellitus in the mother, preterm delivery, fetal distress and macrosomia, failure to progress during the first and second stages of labor, cord prolapse, nuchal cord, true umbilical cord knots, placental abruption, and placenta praevia [15-17].

In the past few decades, we have witnessed a steady rise in global CS rates. In addition to an increase in the numbers of CS deliveries performed worldwide, there has also been a change in the indications for CS; a reflection of changing times [18]. Based on the WHO report for 2007–2008, China had both the highest CS rate (46%) and the highest CDMR rate; the latter accounted for 11.6% of all deliveries in mainland China [5]. Our study shows that these rates have increased even further; Based on our results, the mean rate of CS was 54.90%, while CDMR accounted for 15.53% of all deliveries and 28.43% of the CS deliveries in mainland China. A survey conducted in the US showed that the leading four indications for CS were prolonged labor (dystocia), previous CS delivery, breech presentation, and fetal distress [19]. Unlike the American survey, our study showed that the four leading indications for CS in China were maternal request (28.43%), cephalo-pelvic disproportion (14.08%), fetal distress (12.46%) and previous CS delivery (10.25%). Our results also differed from a survey conducted in a teaching hospital in China in 2013 [14], which showed that the four leading indications for CS were nuchal cord, previous CS delivery, fetal distress and malpresentation [14]. The indications in common among the three surveys were previous CS and fetal distress. Based on the survey conducted by Wang et al. [9], the main indication for CS in 1999 was cephalo-pelvic disproportion, and this changed to previous CS delivery in 2009. That study also showed a significant increase in CS rates from 1999 to 2009, with an increased percentage of CS being performed because of a previous CS [9]. Previous CS is the single greatest risk factor for placenta praevia and placenta accrete. If either of these occurs, there is a risk of catastrophic bleeding at delivery, leading to significant maternal morbidity and mortality. The risk of abnormal placenta rises exponentially with the number of CS deliveries performed, probably as a result of the increasing amount of uterine scar tissue [7]. A survey conducted in a teaching hospital in China showed that the rate of CS because of previous CS increased from 7.22% to 20.9% in 3 years [14]. Similarly, another study showed that previous CS was one of the main indications for performing CS in China (13.6%) [14,19]. In 2006, Tang et al. reported that the percentage of pregnant women with a previous CS delivery increased from 18% in 1992 to 40% in 2000 in urban China [20].

In our study, we found that the main indication for CS was maternal request. With increasing living standards, more women are likely to choose CS as their preferred method of delivery to avoid the issues associated with vaginal delivery, such as the fear of pain during childbirth, subsequent pelvic floor collapse, and incontinence. China’s “one-child policy” was implemented at the end of the 1970s, but was more effective in urban than in rural areas. However, in recent years, there has been some relaxation in the application of the policy all over the country, especially for families in rural areas [14]. Additionally, China instituted a policy this year (2014) to allow more than one child when one of the parents also comes from a single-child family. This may mean that the number of women who will wantmore than one child will increase, and thus, the percentage of pregnant women with a previous CS delivery will increase. Thus, the easing of the one-child policy may translate into an increase in the CS rate.

What are the reasons for the increased CDMR rates among the mainland Chinese population? First, tocophobia (fear of childbirth) may be the most common reason for the increasing rate of CDMR [21]. A survey in 2012 by Pawelec et al. reported that 12% of CS requests by mothers were because of fear of labor pain, and this had increased from a rate of 2% [22]. It has been estimated that 6–10% of all pregnant women have a severe fear of childbirth [23]. Pawelec et al. reported that 52% of pregnant women who had previously requested CS decided on a natural birth after they were informed about methods to reduce labor pain and being guaranteed of the availability of those methods [22]. Therefore, to decrease the rate of CS, appropriate treatment of tocophobia is important.

Second, a common belief in Chinese society, and one reinforced in the media, is that CS delivery is a safer and more convenient way to give birth than vaginal delivery [14]. The perception is that CS affords women a higher level of control over the birth, which they equate with safety and alleviation of fear [24]. This is owed in part to the general perception that CS delivery is much safer now than in the past because of the improvement of the surgical techniques. In addition, there is greater concern among mothers about their subsequent living standard. More women may choose CDMR because of its perceived advantages compared with planned vaginal deliveries, regardless of the potential disadvantages. Vaginal delivery is considered a risk factor for pelvic floor dysfunction, including urinary and anal incontinence, pelvic organ prolapse and sexual dysfunction [25]. It was reported that 26% of primiparous women had urinary incontinence at 6 months postpartum, with the rate being lowest with elective CS (5%), higher with CS during labor (12%), higher still following spontaneous vaginal birth (22%), and highest following vaginal forceps delivery (33%) [26,27]. However, the urinary incontinence rates 2 years after delivery did not differ significantly between planned vaginal and CS births [10,28]. With regard to the safety of the infant, CS was found to be associated with a reduction in the incidence of antepartum stillbirth, brachial plexus injuries related to shoulder dystocia, bone trauma to the clavicle, skull or humerus, intracranial hemorrhage, and neonatal hypoxemic encephalopathy, compared with vaginal delivery [7,10,25,29-31]. Consequently, many women consider CS as the most convenient and safest way to give birth. However, another study reported adverse effects of CDMR on women’s long-term reproductive health [25].

Finally, changes in obstetric management and the increasing autonomy of patients in deciding the mode of delivery may contribute to the increasing rate of CDMR [14]. However, many studies have shown that physicians’ attitudes can significantly influence or motivate patients’ choice of delivery method [14]. A large proportion of obstetricians in the US (46%) [14] and female obstetricians in London (31%) [18] reported that they would favor CS for themselves or for their partners in an uncomplicated pregnancy. In other studies, two-thirds of Turkish obstetricians would prefer CS as the delivery method for themselves or for their partners in an uncomplicated pregnancy [32-35]. Moreover, anxiety of the patient and her family and their insistence on CS was the most commonly stated reason by obstetricians for performing CDMR without any medical indication [32].

However, several surveys have shown that CS has an adverse effect on long-term reproductive health [36], and the potential harm seems to outweigh the benefits.

Therefore, to reduce the rate of CS, we should try to reduce the rate of CDMR. This means that the perception of women and their families that CS is the safest and most convenient way for childbirth needs to be changed. First, appropriate treatment for fear of childbirth is very important. A study showed positive effects of psychoeducational group therapy in nulliparous women with severe fear of childbirth in terms of fewer CS deliveries and more satisfactory delivery experiences relative to control women with a similar severe fear of childbirth [23]. Secondly, as medical personnel, we should explain that the risks of CDMR outweigh the benefits when considering the effects on the woman’s long-term reproductive health, and therefore advocate vaginal delivery as the best method for childbirth. Only in this way can we reduce the rate of CS.

### Strengths and limitations

As a multicenter clinical epidemiological study, we assessed the largest number of deliveries (111,315) from 39 hospitals in 14 provinces and regions over mainland China, while the majority of similar studies assessed a smaller number of deliveries from one hospital or from a local area. This was the major strength of our study.

The lack of information on ethnicity (i.e., Han vs. other ethnicities) and the differences in specialty level of the different hospitals (tertiary vs. secondary vs. primary) are the main limitations. Different results might be obtained for other ethnic groups. Furthermore, the high CS rate issue affects not only China but the whole world. Further analysis about indications for caesarean section in the world should be performed. As a retrospective study, part of the clinical data was not completed and undetected deviations may exist. However, it is important to bear in mind that selection bias and undetected deviations may not have influenced the results. Another limitation of the study was that it was only a descriptive analysis and we did not perform any multivariable analysis.

## Conclusions

Our study results show that CS on maternal request was a considerable driver of the increasing CS rate in mainland China. With the easing of China’s one-child policy, there are likely to be more CS deliveries in the future, because of previous CS delivery. Therefore, to decrease the rate of CS, we should first decrease the rate of CDMR. Appropriate policies and guidelines should be considered to reach this goal.

## Additional file

Additional file 1:
**The procedures of this study received ethics approval from the Human Ethics Committees of following hospitals.**

## Abbreviations

CS: Caesarean section
CDMR: Caesarean delivery on maternal request
