# Supplementary material for: A descriptive analysis of the indications for caesarean section in mainland China
Source: BMC Pregnancy Childbirth. 2014 Dec 12;14:410. doi: 10.1186/s12884-014-0410-2 (PMC4269966; doi:10.1186/s12884-014-0410-2)
Supplement: Additional file 1: — The procedures of this study received ethics approval from the Human Ethics Committees of following hospitals. [file 12884_2014_410_MOESM1_ESM.docx]

**Additional file 1**

The procedures of this study received ethics approval from the Human Ethics Committees of following hospitals

1. Capital Medical University, Friendship Hospital Institutional Review Board
2. Beijing Daxing Maternal and Child Health Hospital Institutional Review Board
3. Tongzhou Maternal and Child Health Hospital Institutional Review Board
4. Inner Mongolia Maternal and Child Health Hospital Institutional Review Board
5. Erlianhaote People’s Hospital Institutional Review Board
6. Taiyuan Maternal and Child Health Hospital Institutional Review Board
7. Cangzhou Central Hospital Institutional Review Board
8. The First Affiliated Hospital of Inner Mongolia Medical University Institutional Review Board
9. Obstetrics and Gynecology Hospital of Fudan University Institutional Review Board
10. Shanghai Changning Maternal and Child Health Hospital Institutional Review Board
11. Shanghai Putuo Maternal and Child Health Hospital Institutional Review Board
12. Nanjing Drum Tower Hospital, The Affiliated Hospital of Nanjing University Medical School Institutional Review Board
13. Wuxi Maternal and Child Health Hospital Institutional Review Board
14. Shandong Provincial Hospital Institutional Review Board
15. Shandong Obstetrics and Gynecology Hospital Institutional Review Board
16. Dongming Country Maternal and Child Health Hospital Institutional Review Board
17. First Affiliated Hospital of Medical College of Xi’an Jiaotong University Institutional Review Board
18. Xi′an Aerospace General Hospital Institutional Review Board
19. Zichang Country People’s Hospital Institutional Review Board
20. Chenggu Country Maternal and Child Health Hospital Institutional Review Board
21. The First Affiliated Hospital of Xinjiang Medical University Institutional Review Board
22. Shengjing Hospital of China Medical University Institutional Review Board
23. Benxi Central Hospital Institutional Review Board
24. Kaiyuan Country People’s Hospital Institutional Review Board
25. Liaohe Youtian Maternal and Child Health Hospital Institutional Review Board
26. Xiuyan Country Maternal and Child Health Hospital Institutional Review Board
27. The Second Hospital Jilin University Institutional Review Board
28. Yushu Maternal and Child Health Hospital Institutional Review Board
29. Nongan Country People’s Hospital Institutional Review Board
30. Hubei Xinhua Hospital Institutional Review Board
31. The Eleventh Hospital of Wuhan Institutional Review Board
32. Wuhan commercial hospital Institutional Review Board
33. The Second Affiliated Hospital of West China Hospital，Sichuan University Institutional Review Board
34. Pengzhou Maternal and Child Health Hospital Institutional Review Board
35. Nanfang Hospital of Nanfang Medical University Institutional Review Board
36. The Third Affiliated Hospital of Nanfang Medical University Institutional Review Board
37. Shaoguan Maternal and Child Health Hospital Institutional Review Board
38. Fuoshan Maternal and the Child Health Hospital Institutional Review Board
